# Gynecomastia: a study to assess how students perceive this disease

**DOI:** 10.3205/iprs000151

**Published:** 2021-02-11

**Authors:** Faisal Ali Al Jabr, Ossama Mohamed Zakaria, Mohammed Ahmed Al Mulhim, Abdulrahman Mohammed Alsuwailim, Hiba AlBurshaid

**Affiliations:** 1 Department of Surgery, College of Medicine, King Faisal University, Al-Ahsa, Eastern Province, Saudi Arabia; 2 University of Dammam, Saudi Arabia

**Keywords:** King Faisal University, Saudi Arabia, knowledge, perception, gynecomastia

## Abstract

**Background**: Gynecomastia is a benign proliferation of the glandular male breast tissue. Gynecomastia etiology might be physiological or non-physiological such as medications, chronic diseases (e.g. hypogonadism), or steroid supplements.

**Aim**: The purpose of this study was to assess the knowledge and understanding of gynecomastia among medical students and which resources were used to gain their understanding regarding the disease.

**Methods**: Data for this qualitative, questionnaire-based cross-sectional study was collected on the basis of our own study objectives and from available questionnaires with similar objectives. The questionnaire was composed of 26 questions divided into many items that were recorded including sociodemographic data, gynecomastia symptoms, and holistic perception of the problem by the students. Exclusion criteria included those who refused to participate in the study and did not complete the questionnaire. Statistical tests were taken significant at p-value ≤0.05. All analyses were performed using SPSS, version 21.

**Results**: A total of 200 medical students participated in this study, among them more males than females (64% vs. 36%). We observed that medical students had significantly more moderate knowledge with teachers as their source of information on gynecomastia (p=0.028) while with books (p=0.005) and internet (p=0.041) as their sources of information they had significantly more a higher level of knowledge.

**Conclusions**: Medical students have overall insufficient knowledge about gynecomastia especially in physical examination and treatment aspects. Therefore, gynecomastia is to be considered more thoroughly in the curriculum.

## Zusammenfassung

**Hintergrund**: Gynäkomastie ist eine gutartige Wucherung des Drüsengewebes der männlichen Brust. Gynäkomastie kann physiologische oder nicht-physiologische Ursachen haben, also beispielsweise durch Medikamente, chronische Krankheiten (z.B. Hypogonadismus) oder Steroidpräparate ausgelöst werden.

**Ziel**: Ziel dieser Studie war es, Kenntnisse und Verständnis der Gynäkomastie unter Medizinstudenten zu beurteilen und zu ermitteln, welche Ressourcen zur Erlangung dieser genutzt wurden.

**Methoden**: Die Daten für diese qualitative, Fragebogen-basierte Querschnittsstudie wurden auf der Grundlage unserer Studienziele und verfügbarer Fragebögen mit ähnlichen Zielen erhoben. Der Fragebogen bestand aus 26 Fragen, die in mehrere Items unterteilt waren, die u.a. soziodemographische Daten, Gynäkomastie-Symptome und ganzheitliche Wahrnehmung des Problems der Studenten erfassten. Zu den Ausschlusskriterien gehörten diejenigen, die sich weigerten, an der Studie teilzunehmen und den Fragebogen nicht ausfüllten. Statistische Tests wurden bei einem p-Wert ≤0,05 als signifikant angesehen. Alle Analysen wurden mit SPSS, Version 21, durchgeführt.

**Ergebnisse**: Insgesamt nahmen 200 Medizinstudenten an dieser Studie teil, darunter mehr Männer als Frauen (64% vs. 36%). Wir beobachteten, dass Medizinstudenten mit Lehrern als Informationsquelle über Gynäkomastie signifikant eher mäßiges Wissen hatten (p=0,028), während sie mit Büchern (p=0,005) und Internet (p=0,041) als Informationsquellen signifikant einen höheren Wissensstand hatten.

**Schlussfolgerungen**: Medizinstudenten haben insgesamt ein unzureichendes Wissen über die Gynäkomastie, insbesondere in Bezug auf die körperliche Untersuchung und die Behandlung. Deshalb sollte die Gynäkomastie im Curriculum gründlicher berücksichtigt werden.

## Introduction

The breast is defined as a modified sweat gland. It is an organ that is embedded in a fatty matrix, along with lymphatics and blood vessels [1]. The most common male breast lesion is gynecomastia. It is defined as a benign proliferation of the glandular male breast tissue [2]. It can present within different age groups, from newborns to older men [3]. The causes of gynecomastia may either be physiological or non-physiological. Physiological causes of gynecomastia do not require further treatment since gynecomastia may resolve on its own. However, the non-physiological type may be induced by certain proven medications, pathologies like hypogonadism, steroid supplement medications and tumors [4]. 

Ductal epithelial hyperplasia along with ductal elongation and branching, with increase in vascularity is influenced by estrogen. Estrogen acts as a growth precursor for the male breast enlargement [2]. The mechanism behind the development of gynecomastia is due to hormonal imbalance. There is elevation of estrogen and/or testosterone as well [5]. 

On the one hand, many factors may lead to an increase of estrogen levels such as direct secretion (from testes/adrenals/placenta), extra-glandular aromatization of precursors, decreased metabolism and exogenous administration. On the other hand, testosterone levels are decreased due to glandular inhibition, increase metabolism and increase binding to sex hormone-binding protein [2]. 

Proper clinical evaluation of patients with gynecomastia is crucial to rule out similar conditions like pseudogynecomastia, which most frequently occurs in obese men with fat deposition giving the appearance of a female breast rather than a proliferation of the glandular tissue. Moreover, it is also seen in patients with massive weight loss [2], [6]. 

The main objectives of treating gynecomastia are alleviation of the underlying cause, reliving the discomfort, restoring the normal shape and reassurance regarding outcome. 

Management of gynecomastia depends on the etiology of gynecomastia. If being drug-induced, it will be managed by stoppage of drug. Yet, if due to a chronic illness, it will be relieved by treating the underlying cause [2]. The aim of the current qualitative questionnaire-based study is to evaluate the knowledge and perception of medical students of the holistic aspects of the gynecomastia problem. 

## Methodology

The ethical considerations were fulfilled through the approval of the ethical committee. Data confidentiality was maintained through the whole study.

This is a qualitative, questionnaire-based cross-sectional study. An anonymous questionnaire was distributed to a random sample of 200 medical students from 4 national medical schools. Questions were designed to evaluate the students’ knowledge level and attitudes towards the problem of gynecomastia. The level of knowledge about gynecomastia of medical students was assessed using 18 questions as described in Table 1 (Tab. 1). The study population included medical students registered in the first academic year up to those in the final year of medical school. The colleges included in the study were from Al-Ahsa (King Faisal University), Dammam (Imam Bin Abdulrahman University) and Al Majma’ah University. Many items were recorded including socio-demographic data, gynecomastia symptoms, and holistic perception of the problem by the students. Data were collected through Google Sheet of a specially designed questionnaire [7]. Exclusion criteria included those who refused to participate in the study and did not complete the questionnaire. 

All identified correct answers were marked and coded as 1 while the incorrect answers were coded as 0. A score range from 0 to 26 was obtained and the cutoff points of 50% and 75% of the total score points were used to determine low, moderate and high knowledge. Medical students’ knowledge was classified as low by the score range of 0 to 10 points, as moderate by the score range of 11 to 17 points and as high by the score range of 18 to 26 points. 

## Statistical analysis

Both descriptive and inferential statistics were performed for data analysis. Descriptive statistics were presented using number and percentages. Statistical analysis was carried out using SPSS Statistics, version 21.0. Statistical tests were considered significant at p-value ≤0.05. The relationship between determinants of knowledge and academic year level was examined using Chi-square test.

## Results

**Figure 1:**
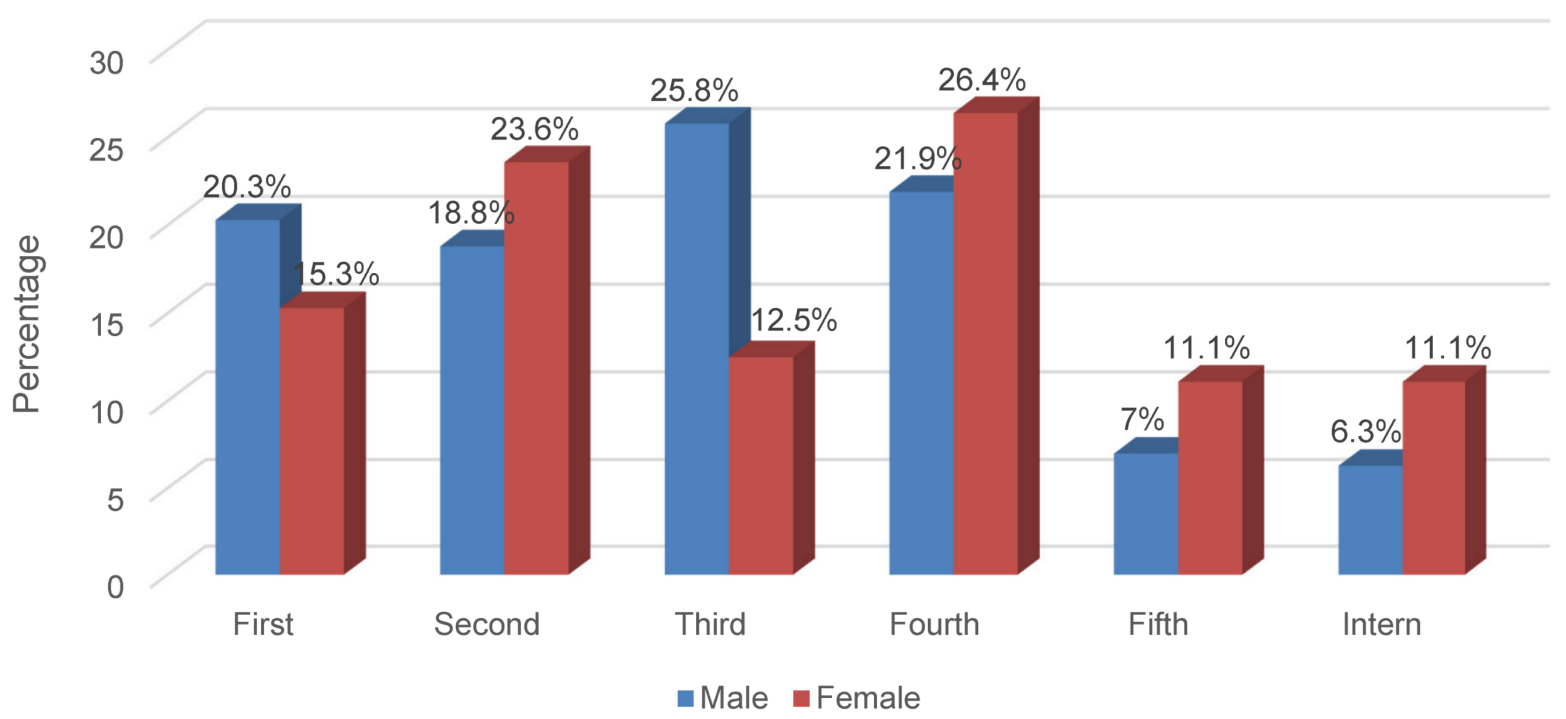
Academic year level in relation to gender

A total of 200 medical students was recruited for the study. Table 2 (Tab. 2) represents the sociodemographic characteristics of the participants. More males than females participated in the study (64% vs. 36%). The majority of the participants was in fourth year level (23.5%), followed by third year (21%) and second year level (20.5%) (Table 2 (Tab. 2)). Comparing gender and academic year level, the majority of the males was in third year level (25.8%) while most of the females were in fourth year level (26.4%) (Figure 1 (Fig. 1)).

The prevalence of gynecomastia among the participants was 3.5% with most cases in the junior level (p=0.084) (Table 3 (Tab. 3)). Seventeen of the medical students stated that they have a relative or friend who has been diagnosed with gynecomastia, with significantly more cases in the junior level group (p=0.038). Additionally, poor perception was found among medical students with regards to the statement about “approaching a relative or friend who suspects having gynecomastia”, with junior level more would approach (p=0.579).

The determinants of knowledge about gynecomastia have been described in Table 1 (Tab. 1). It was revealed that the majority of medical students (73%) knows that the breast is considered as exocrine gland. Nearly all (93%) were aware that gynecomastia is a swelling of the breast tissue in boys or men. Regarding the cause of gynecomastia, most of them (85%) were aware of its cause and knew that it is more to men (83.0%) than women (2.5%). A high proportion of the students (74%) correctly answered that a swollen breast gland tissue is one of the symptoms of gynecomastia. The majority (90%) knew that gynecomastia is not an infectious disease. More than half of the students (54.5%) were unaware that gynecomastia can affect one breast or both breasts, and 84.5% of them did not know that the prevalence of gynecomastia peaks again at the age of 50 to 69. Eighty-eight percent of the students lacked the knowledge that galactorrhea is not one of the presentations of gynecomastia. Asked for the drugs that increase the risk of gynecomastia, students answered as follows:

- hormones (49%) including anabolic steroids, growth hormones and estrogen,
- androgen receptor blockers (21.5%) such as flutamide, spironolactone, cimetidine and marijuana,
- psychiatric drugs (17.5%) like diazepam, haloperidol and tricyclic antidepressants,
- gastrointestinal agents (10%) such as Cimetidine, Ranitidine and Omeprazole,
- illicit drugs (8%): alcohol, heroin, marijuana, methadone and amphetamines,
- antibiotics (4%): Isoniazid, Ketoconazole and Metronidazole,
- antihypertensives (3.5%): Verapamil, Enalapril and Amlodipine.

A high number of students (79.5%) either answered incorrectly or did not know that using illegal drugs cannot help in preventing gynecomastia. Sixty-three percent were not aware that an enlargement of the breast with fat tissue cannot be called gynecomastia. Regarding the physical examination, there were 71% who did not know that on physical examination, the physician will feel a disc or firm tissue that is concentric with the nipple-areolar complex, and 81.5% did not know that for patients with enlarged fatty breast, the resistance to fingers will meet until they reach to nipple. 

In terms of treatments, students think that the most common way to treat gynecomastia is to stop medications that cause gynecomastia (20%), followed by the use of medication (14%) and surgery (15%). Medical students had poor knowledge about Anastrozole as 70.5% of them indicated no knowledge on what the medication should be categorized. Furthermore, 82.5% of the students exemplified poor knowledge about gynecomastia among adolescents and whether they have to undergo surgery before testes reach adult size. For surgical treatment options, only 14.5% knew that both subcutaneous mastectomy and subcutaneous mastectomy with liposuction are applicable options, while 67.5% had no idea about these surgical options.

Following the comparison of academic year level versus the determinants of knowledge, we found that interns had significantly better knowledge that gynecomastia is a swelling of the breast tissue in boy or men (p=0.036). We also found that interns were significantly more knowledgeable that swollen breast gland tissue is one of the symptoms of gynecomastia (p=0.001), that gynecomastia is not an infectious disease (p=0.003) and that the prevalence of gynecomastia peaks between the ages of 50 and 69 (p=0.049). However, a significantly poor knowledge was shown on junior level with regards to the statement that a patient with gynecomastia may not come with galactorrhea (p=0.002). With regards to the relationship of knowledge about drugs that increased the risk of gynecomastia and the year level, we observed that interns had significantly better knowledge that hormonal treatment (p<0.001), androgen receptor blockers (p=0.005), GI agents (p<0.001) and antibiotics (p=0.014) could increase the risk of gynecomastia. In addition, interns showed significantly better knowledge that the physician would feel a disc or firm tissue that is concentric with nipple-areolar complex (p=0.014). However, senior level showed significantly better knowledge that stopping medications is a treatment for gynecomastia (p=0.006) and that the use of other medications is another option (p=0.040). Furthermore, junior level showed significantly low knowledge that surgery should not be recommended to adolescents (p=0.010) and that subcutaneous mastectomy is recommended for gynecomastia (p=0.038) while interns showed significantly better knowledge that subcutaneous mastectomy with liposuction should be recommended as a surgery for gynecomastia (p=0.037).

**Figure 2:**
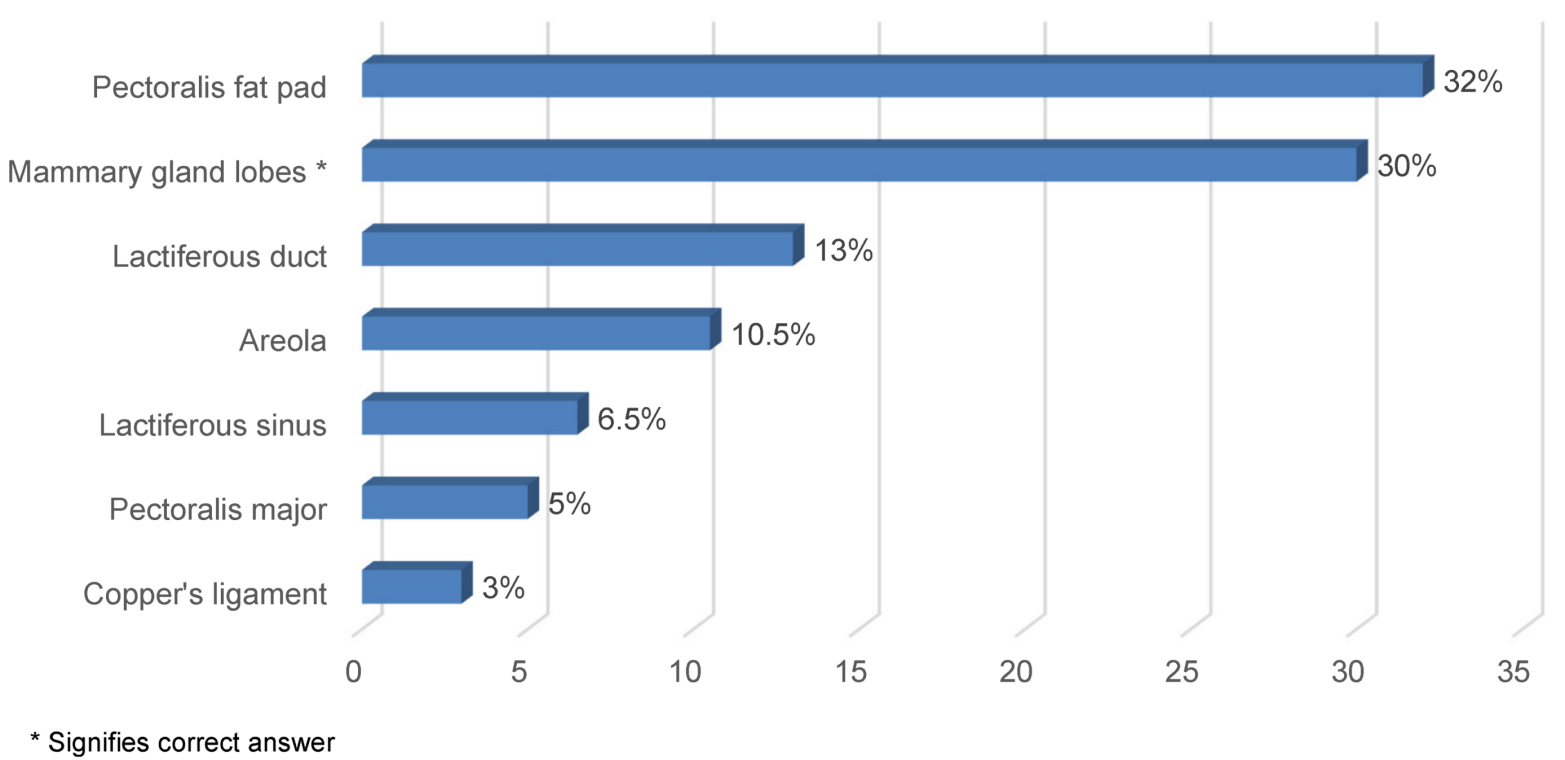
Knowledge on the anatomical components of the breast affected by gynecomastia

Figure 2 (Fig. 2) presents the knowledge about the anatomical components of the breast affected by gynecomastia. It was revealed that Pectoralis fat pad (32%) was the most commonly known anatomical component affected by gynecomastia, followed by mammary gland (30%) and lactiferous duct (13%).

**Figure 3:**
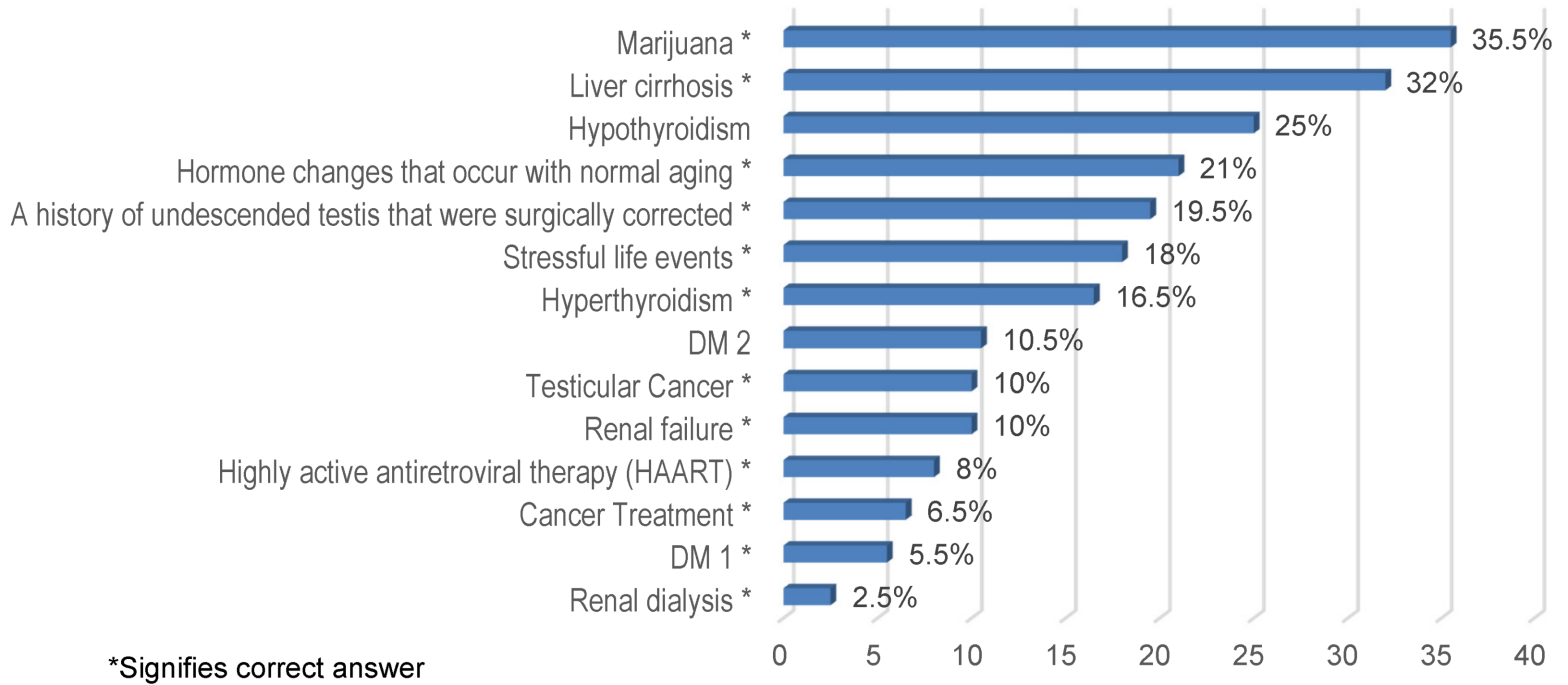
Knowledge on the causes of gynecomastia

Figure 3 (Fig. 3) shows the knowledge about the causes of gynecomastia. Based on the results, marijuana (35.5%) was the most commonly known cause of gynecomastia, followed by liver cirrhosis (32%), whereas renal dialysis (2.5%) was the least known.

**Figure 4:**
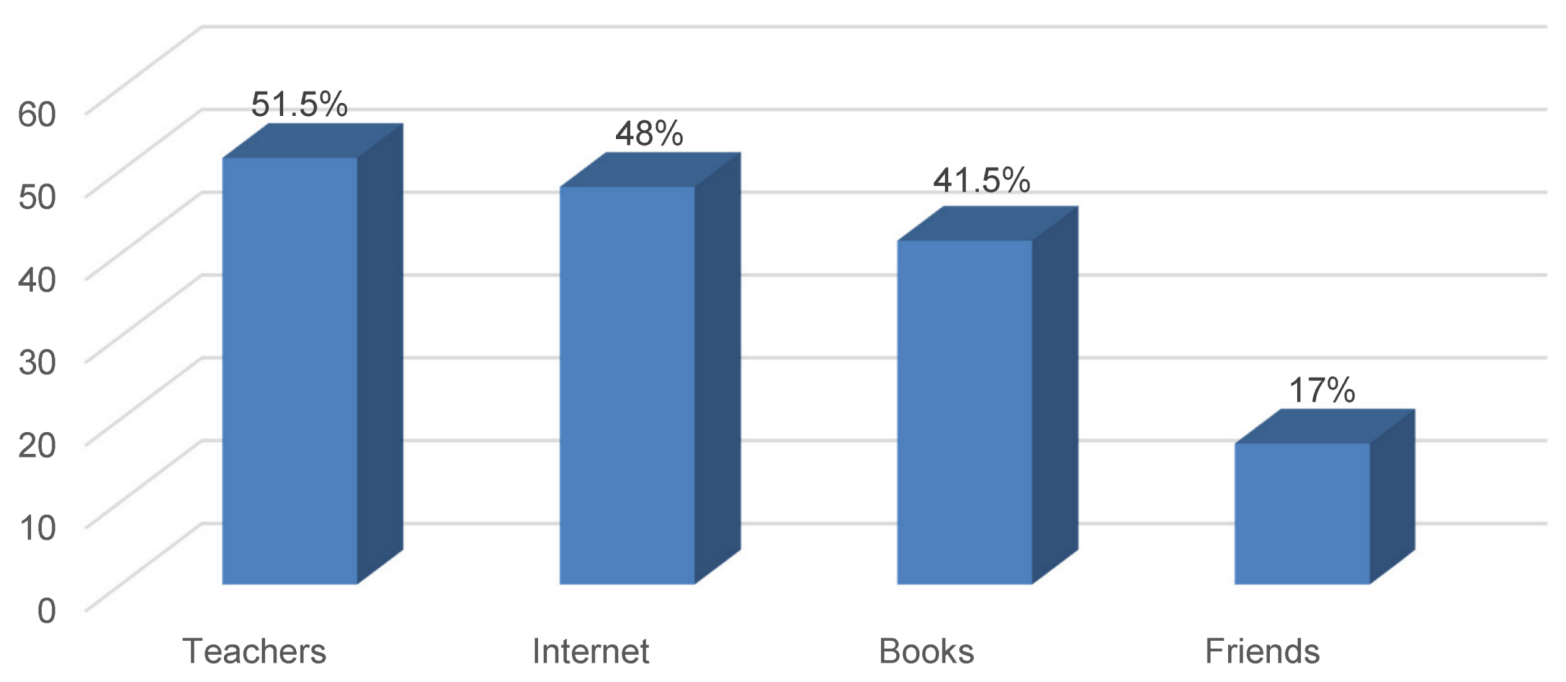
Sources of information

The most common sources of information regarding gynecomastia were teachers (51.5%), followed by internet (48%) and books (41.5%) (Figure 4 (Fig. 4)).

Table 4 (Tab. 4) describes the prevalence of knowledge on gynecomastia. Based on the results, the mean knowledge score obtained from the determinants of knowledge was 11.4 (SD 5.16) out of 26 points and based on cutoff points to determine, low, moderate and high knowledge (50% and 70% from the total score points). Low, moderate and high knowledge were accounted to 49%, 37% and 14% of participants, respectively.

When measuring the relationship between the level of knowledge and gender, academic year level and sources of information, it was found that junior students had significantly more a low level of knowledge (p<0.001). The most common source of information regarding gynecomastia was teachers, followed by internet and books while the least used of them was friends. We also observed that medical students had significantly more a moderate knowledge with teachers as their source of information on gynecomastia (p=0.028) while with books (p=0.005) and internet (p=0.041) as sources of information they had significantly more a higher level of knowledge. Gender did not show a significant relationship with the level of knowledge (Table 5 (Tab. 5)).

When comparing the academic year level and the sources of information, it was found that junior level was significantly less associated with books as their sources of information (p<0.001) while they positively associated with internet for finding information about gynecomastia (p=0.015). Although junior levels did not frequently ask teachers for information on gynecomastia, the result did reach statistical significance (p=0.079). Interns the most did not rely on friends as their sources of information on gynecomastia, however, this did not differ significantly (p=0.481) (Table 6 (Tab. 6)).

## Discussion

Gynecomastia is defined as a benign proliferation of the glandular male breast tissue [2], [3]. It is one of the common lesions of the male breast affecting at least 30% of males at some point during their life [2]. The databases that were used for the search of literature were PubMed, Scopus, Science Direct and Cochran Library; the words “Gynecomastia”, “Awareness”, “Perception”, and “Medical students” were used in the search. However, the databases have yielded zero results regarding researches about gynecomastia perception among medical students. To our knowledge, the current study is the first to evaluate gynecomastia perception among any population section. 

Gynecomastia etiology might be physiological or non-physiological such as proved medications, pathological diseases (e.g. hypogonadism), steroid supplements and certain tumors [8]. Different studies revealed that drug-induced gynecomastia has been attributed to the following [2], [3]:

1. androgen receptor blockers: Flutamide, Spironolactone, Cimetidine and Marijuana,
2. antibiotics: Isoniazid, Ketoconazole and Metronidazole,
3. antihypertensives: Verapamil, Enalapril and Amlodipine,
4. GI agents: Cimetidine, Ranitidine and Omeprazole,
5. illicit drugs: alcohol, heroin, marijuana, methadone and amphetamines,
6. psychiatric drugs: Diazepam, haloperidol and tricyclic anti-depressants.

However, our study population has shown a lack of knowledge regarding the drugs that are attributed to gynecomastia development; with antihypertensives being least among other medications, and androgen receptor blockers being highest in the list. This tells us that our study population needs to focus more on gaining knowledge in this aspect of gynecomastia development. 

Proper clinical evaluation of patients with gynecomastia is crucial to rule out similar conditions like pseudogynecomastia, which most frequently occurs in obese men with massive weight loss and in obese men with fat deposition, hence resembling a female breast, rather than a proliferation of the glandular tissue [6]. However, the population we have studied revealed a lack of understanding that enlargement of the breast with fat tissue cannot be called gynecomastia. Moreover, it also revealed a lack of knowledge that the physician will feel a disc or firm tissue that is concentric with the nipple-areolar complex during the examination. 

The management of gynecomastia is based on the underlying cause for its development, for example if drug-induced then it should be stopped [9]. If due to chronic illness, it will be relieved by treating the underlying cause [2]. Knowledge on treatment aspect of gynecomastia was not satisfactory. Medical students had a lack of knowledge regarding the need to stop the attributed drugs as well as the necessity to treat the underlying cause of gynecomastia. This yields an important issue of medical students to have to know the medications taken by the patients from the history as well as whether the patients have chronic diseases that are attributed for the development of gynecomastia.

Surgical treatment options being applicable for gynecomastia are both subcutaneous mastectomy and subcutaneous mastectomy with liposuction [10]. However, the majority of our study population revealed no single idea what are the surgical options for gynecomastia. In our study, this tells us that aspect of gynecomastia is important to be covered by medical students during the studies. 

Consequences of gynecomastia may include fear of malignancy, anxiety, depression, social phobias, low self-esteem, disordered eating, being dissatisfied of being a male, reduced quality of life, maladaptive coping mechanisms and body image concerns [2], [11], [12], [13], [14].

Knowledge and information sources were mainly obtained from references and data-based evidences. Students who scored high answered that books and internet equally are their most common sources of information whereas students who scored low rely mostly on internet as source of information. This tells us how important adding textbooks into students’ sources is to obtain solid and correct information regarding this condition.

### Limitations and recommendations

This study is limited by low participants, further studies on larger group of medical students and general population are recommended.

## Conclusion

Medical students have overall insufficient knowledge about gynecomastia especially in physical examination and treatment aspects. Medical students who scored high most commonly use textbooks and internet equally as their source of information. Students who scored low rely mostly on internet to gain their knowledge. Therefore, students should be encouraged more to add textbooks into their primary information sources. Also, gynecomastia is to be considered more thoroughly in the curriculum.

## Notes

### Competing interests

The authors declare that they have no competing interests.

## Figures and Tables

**Table 1:**
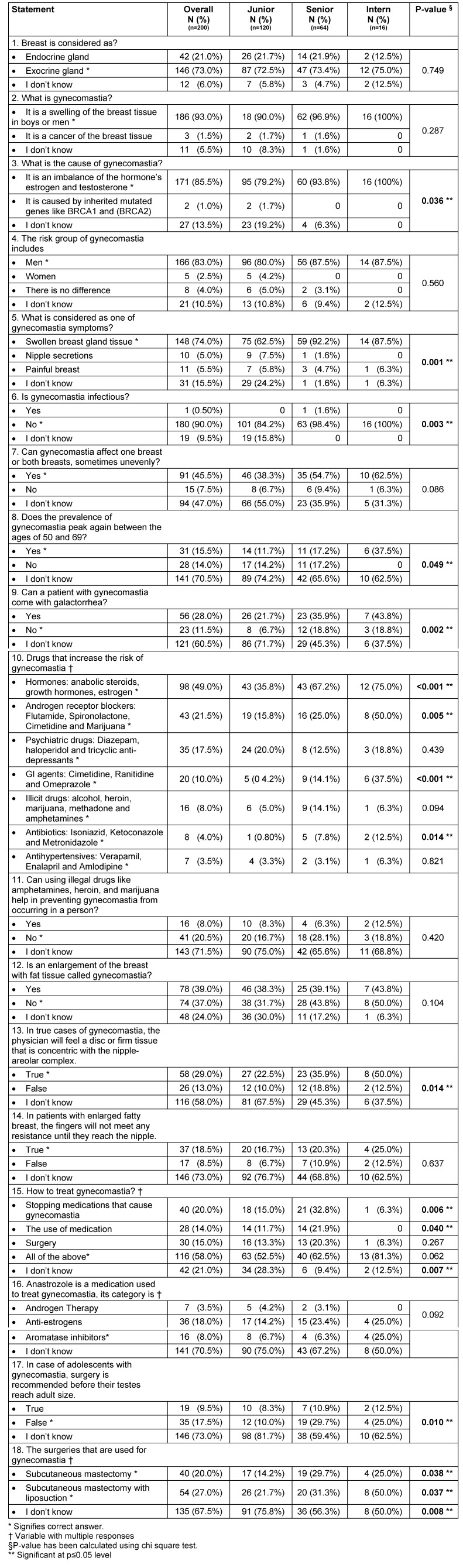
Determinants of knowledge in relation academic year level

**Table 2:**
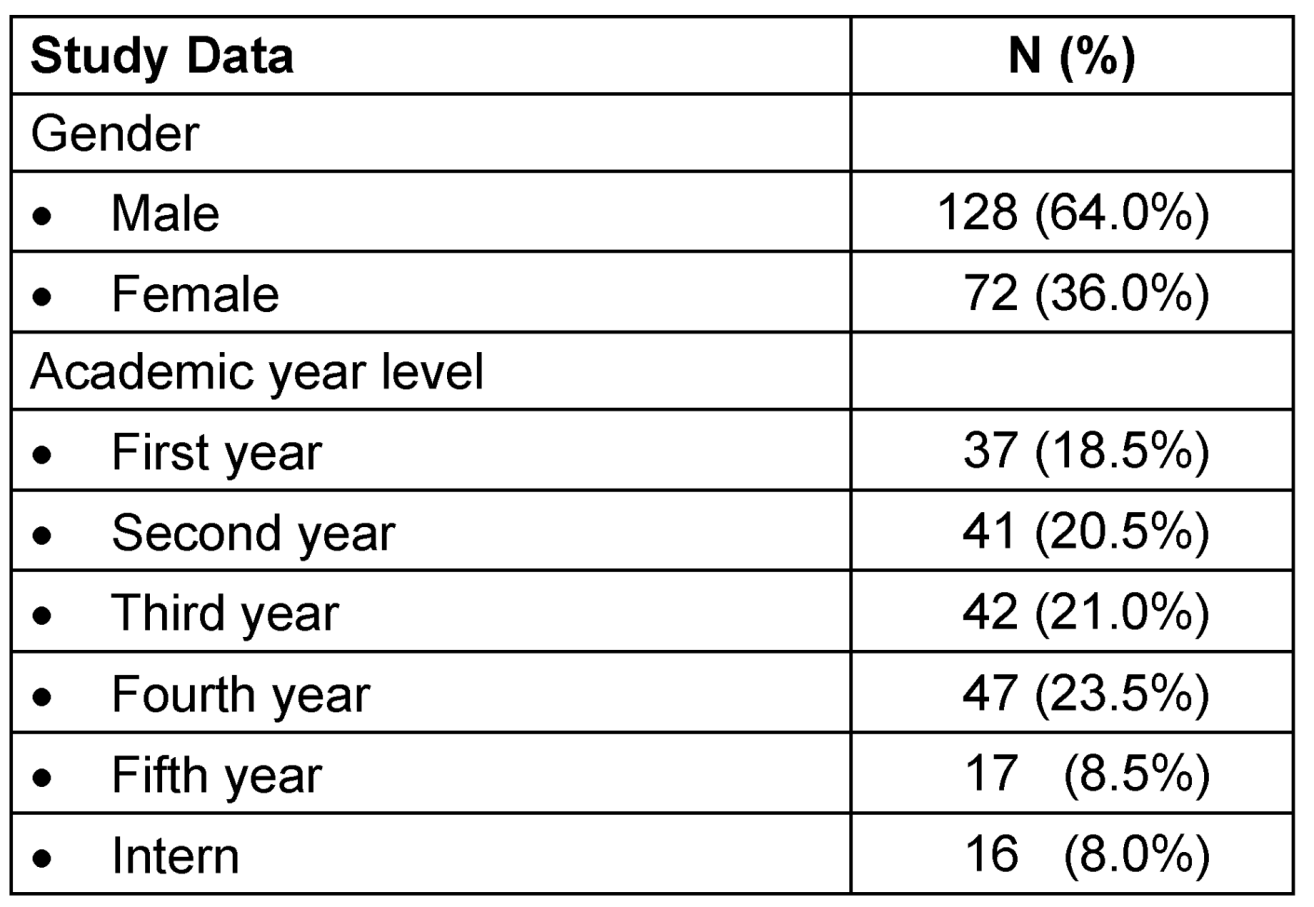
Sociodemographic characteristics of the participants ^(n=200)^

**Table 3:**
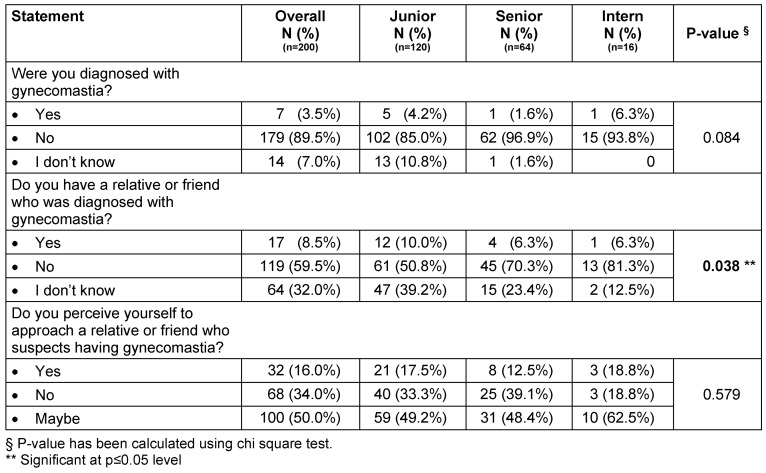
Prevalence of gynecomastia in relation to academic year level

**Table 4:**
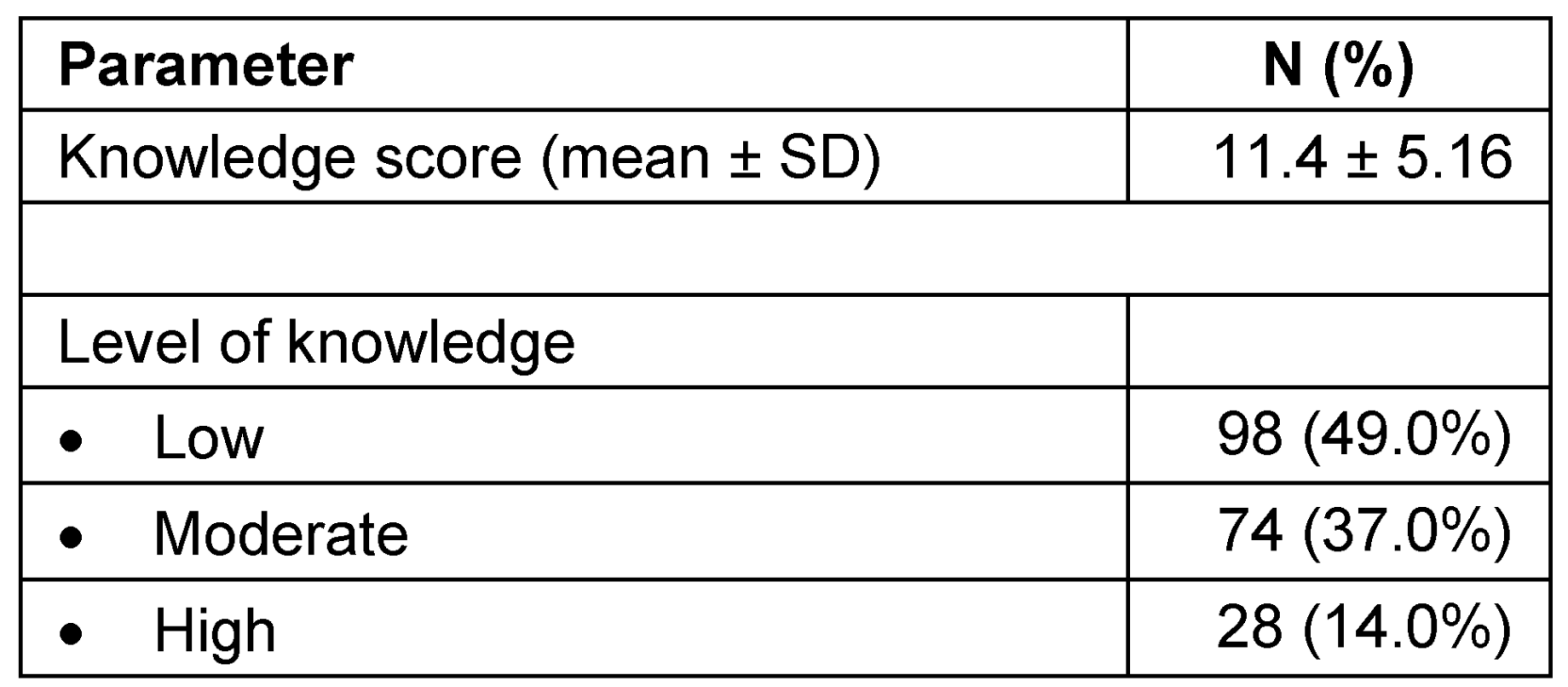
Prevalence of knowledge about gynecomastia ^(n=200)^

**Table 5:**
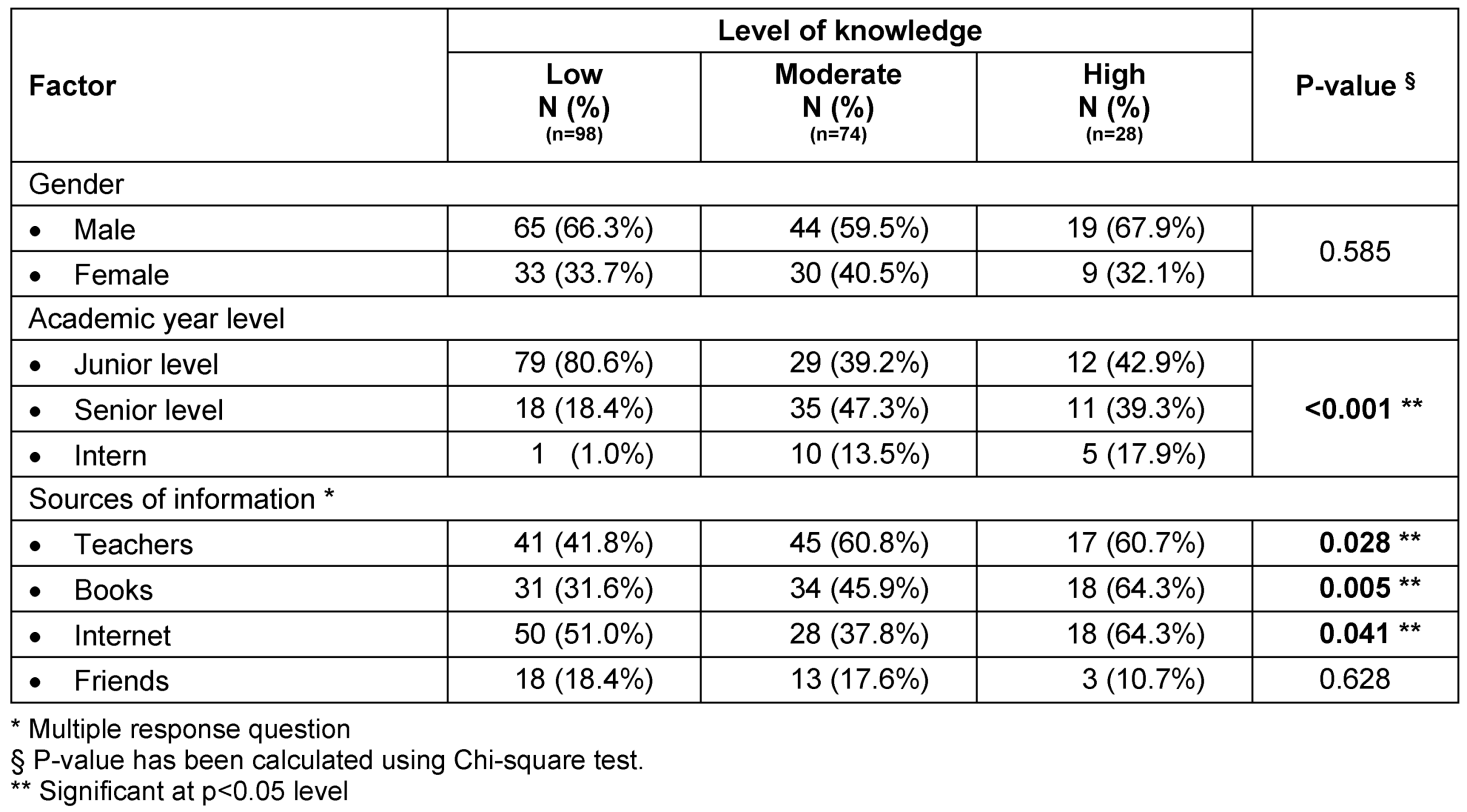
Determining the factor associated with knowledge about gynecomastia ^(n=200)^

**Table 6:**
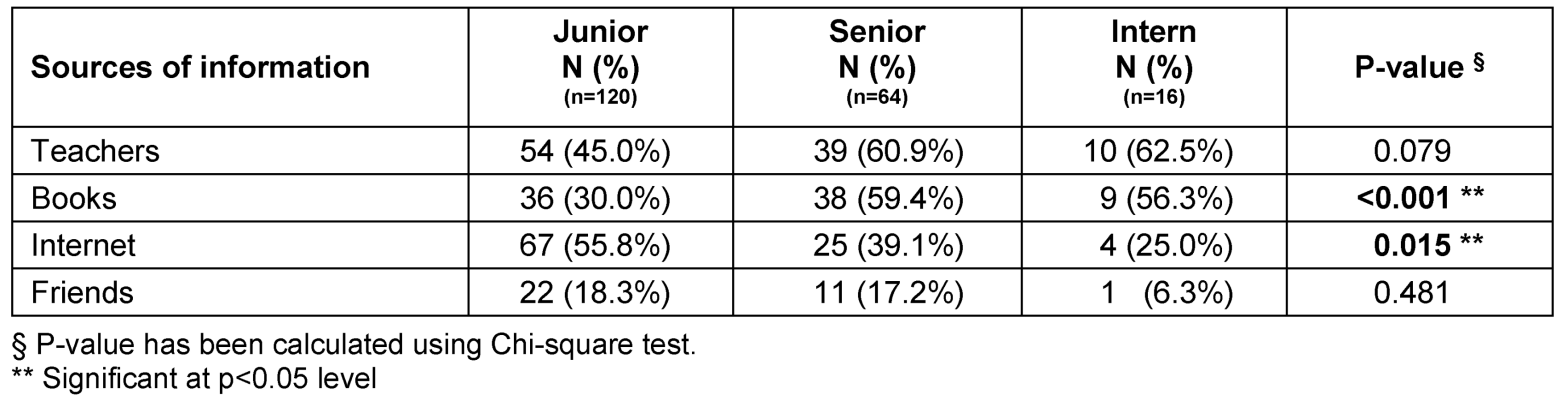
Relationship between the sources of information and the academic year level
